# Using Stigmergy to Distinguish Event-Specific Topics in Social Discussions

**DOI:** 10.3390/s18072117

**Published:** 2018-07-02

**Authors:** Mario G. C. A. Cimino, Alessandro Lazzeri, Witold Pedrycz, Gigliola Vaglini

**Affiliations:** 1 Department of Information Engineering, University of Pisa, 56122 Pisa, Italy; alessandro.lazzeri@for.unipi.it (A.L.); gigliola.vaglini@unipi.it (G.V.); 2 Department of Electrical and Computer Engineering, University of Alberta, Edmonton, AB T6G 2G7, Canada; wpedrycz@ualberta.ca

**Keywords:** microblog analysis, time series similarity, stigmergy, term cloud, receptive field

## Abstract

In settings wherein discussion topics are not statically assigned, such as in microblogs, a need exists for identifying and separating topics of a given event. We approach the problem by using a novel type of similarity, calculated between the major terms used in posts. The occurrences of such terms are periodically sampled from the posts stream. The generated temporal series are processed by using marker-based stigmergy, i.e., a biologically-inspired mechanism performing scalar and temporal information aggregation. More precisely, each sample of the series generates a functional structure, called mark, associated with some concentration. The concentrations disperse in a scalar space and evaporate over time. Multiple deposits, when samples are close in terms of instants of time and values, aggregate in a trail and then persist longer than an isolated mark. To measure similarity between time series, the Jaccard’s similarity coefficient between trails is calculated. Discussion topics are generated by such similarity measure in a clustering process using Self-Organizing Maps, and are represented via a colored term cloud. Structural parameters are correctly tuned via an adaptation mechanism based on Differential Evolution. Experiments are completed for a real-world scenario, and the resulting similarity is compared with Dynamic Time Warping (DTW) similarity.

## 1. Introduction

Microblogging is a broadcast process that allows users to exchange short sentences. These short messages are important sources of information and opinion about socially relevant events. Microblogging systems such as Twitter, Tumblr, and Weibo are increasingly used in everyday life. Consequently, a huge number of informal, unstructured messages are produced in real time. In the literature, a research challenge is to identify and separate the topics of a specific event.

**Figure 1:**
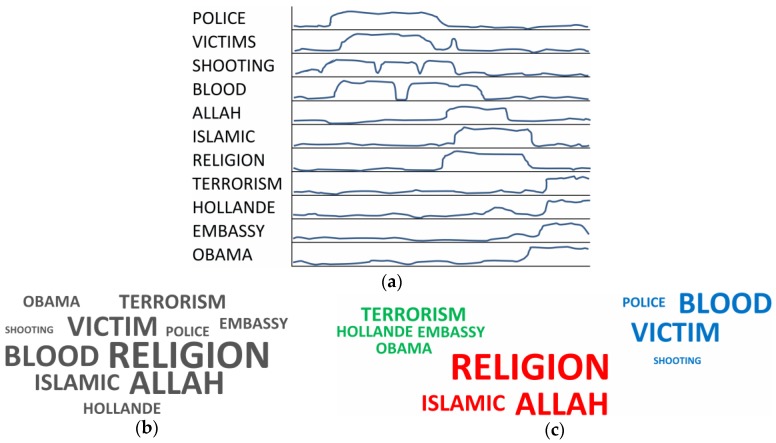
(**a**) Terms and temporal series; (**b**) Term Cloud; (**c**) Relational Term Cloud.

A commonly encountered approach is to adopt visual representations to summarize the content of the messages [1,2,3,4,5,6,7]. A term cloud is a straightforward means to represent the content of the discussion topics of a given event [8]. This is achieved by arranging the most frequent terms at the center of the term cloud, and positioning less frequent ones on the border, by using a font size proportional to the frequency [9]. In practice, a conventional term cloud does not include information about the relationship between terms. The purpose of this study is to enrich the cloud representation to generate a relational term cloud, by exploiting a similarity measure between terms. Figure 1a shows a sub-list of major terms used on Twitter during the terrorist attack in Paris on 13 November 2015, by gunmen and suicide bombers. For each term, a simplified example of time series segment, generated by the term occurrences, and periodically sampled as scalar value, is also represented. Here, it can be noticed that the terms *police*, *victim*, *shooting* and *blood*, related to the story, are mostly used in the first part of the event. In contrast, the terms *Allah*, *Islamic*, *religion*, related to the religious topic, are mostly used in the middle. Finally, the terms *terrorism*, *Hollande*, *embassy,* and *Obama*, related to politics, are used at the end. Figure 1b shows a conventional term cloud, generated based on the global occurrences of each term.

Figure 1c shows a relational term cloud, generated via a clustering process based on a similarity measure between terms. Here, terms belonging to different discussion topics are represented with a different color. Several time series segments may generate consecutive relational term clouds, showing also the temporal evolution of the major topics. More specifically, in our approach the similarity between two terms is the similarity of the related time series. This means that the terms used in the same discussion topic have similar usage over time, i.e., similar time series [10]. In the literature, the term similarity is usually derived from the co-occurrence of the terms in the same text message [1,10], which is the special case of identical time series. In contrast, our notion of similarity between the time series generalizes the co-occurrence, by considering other fundamental processes of discussion topics such as the scalar and temporal proximity between terms of different participants. Moreover, our notion of similarity deals with different types of noise, such as ripple, amplitude scaling, drift, offset translation, and temporal drift [11].

**Figure 2:**
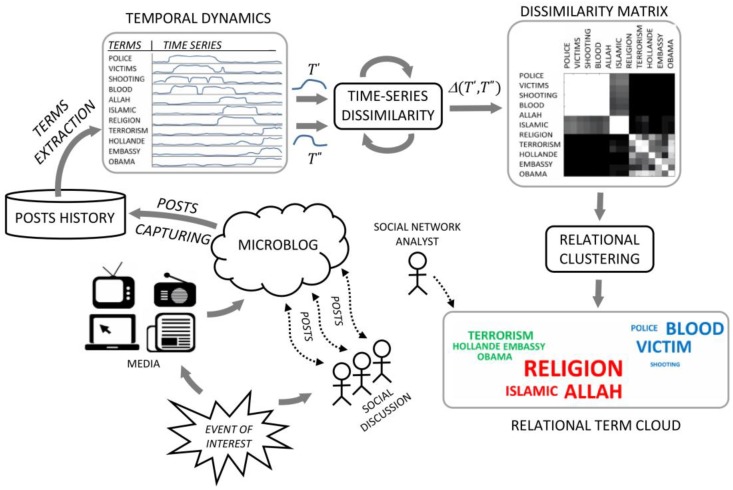
Overview of the proposed approach.

Figure 2 shows an overall representation of the proposed approach. Here, the starting point is an event of social interest, directly observed by humans or delivered via traditional or modern communication media. Opinions and facts are then posted to the microblog while the event unfolds. The first step of the social sensing process is performed by posts capturing, which takes the stream of recent or current messages related to the event and feeds the posts history storage. The major terms are then extracted from the posts history together with their temporal dynamics. Such time series are used as a basis for calculating the co-occurrence of terms, determining a similarity measure between 0 and 1. For better clarity and correspondence to conventional distance measures, similarity is often provided in terms of its complement, i.e., the dissimilarity measure, which takes two input time series windows, $T^{\prime }$ and $T^{\prime \prime }$, and returns the output dissimilarity value, $\Delta (T^{\prime },T^{\prime \prime })\in [0,1]$. A dissimilarity matrix is then created by applying the dissimilarity measure to all pairs of time series windows of the current major terms. Given, the dissimilarity matrix, the purpose of the relational clustering algorithm is to generate different clusters of terms, each characterizing a different social discussion topic. The different clusters are finally represented as a relational term cloud.

To compute the similarity between time series we adopt marker-based stigmergy, i.e., a biologically inspired mechanism performing scalar and temporal information aggregation. Stigmergy is an indirect communication mechanism that occurs in biological systems [12]. In computer science, marker-based stigmergy can be employed as a dynamic, agglomerative, computing paradigm because it embodies the time domain. Stigmergy focuses on the low-level processing, where individual samples are augmented with micro-structure and micro-behavior, to enable self-aggregation in the environment. Self-aggregation of data means that a functional structure appears and stays spontaneous at runtime when local dynamics occur.

The proposed mechanism works if structural parameters, such as the mark attributes, are correctly tuned. For example, a very large and persistent mark may cause growing trails with no stationary level, because of a too dominant and long-term memory effect. A very small and volatile mark may cause poor mark aggregation. For tuning such parameters, we adopt an adaptation mechanism based on Differential Evolution (DE). DE is a stochastic optimization algorithm based on a population of agents, suitable for numerical and multi-modal optimization problems. In the last decades, DE has been applied to many applications and research areas, including parameterization of stigmergy-based systems [11]. DE exhibits excellent performance both in unimodal, multimodal, separable, and non-separable problems, when compared with other similar algorithms, such as genetic algorithms and particle swarm optimization. 

Overall, in this paper, a novel technique is explored to realize a similarity measure between term occurrences in event-specific social discussion topics. The similarity measure is based on stigmergy as a vehicle of both scalar and temporal proximity and exploits Self-Organizing Maps to carry out a clustering between terms. To manage the considerable level of flexibility offered by stigmergy in recognizing different patterns, we propose a topology of multilayer network based on Stigmergic Receptive Fields (SRFs). We discuss in detail a design strategy of the novel architecture that fully exploits the modeling capabilities of the contributing SRFs.

To study the effectiveness of the proposed approach, we applied it to analyze the discussion topics occurred on Twitter during the November 2015 Paris terrorist attack. We compared the quality of the stigmergic similarity with a popular distance measure used for time series, i.e., the Dynamic Time Warping (DTW) distance [13,14].

The paper is structured as follows: Section 2 covers the related work. Section 3 and Section 4 focus on the proposed architecture. Section 5 presents case studies and comparative analysis. Section 6 draws the conclusion. Finally, Appendix A shows the parameterization of the proposed system.

## 2. Related Work

Several research works have been developed in recent years with the aim of exploiting information available on social media to derive discussion topics. Such works either describe working systems or focus on a single specific challenge to be studied. Thus, the systems available on the literature present different degrees of maturity. Some systems have been deployed and tested in real-life scenarios, while others remain under development. Among the proposed systems some approaches are tailored to suit requirements of a specific kind of event and are therefore domain specific. Thus, a comparative performance evaluation is not feasible due to the heterogeneity of goals, methodologies, and events. In this section, the relevant works in the field are summarized, discussing differences and similarities with our approach.

The approaches for determining the topic of microblogs differ in terms of number of posts considered, use of external resources, methodology, topic representation. Similarity measures can be based on external resources, such as WordNet and Wikipedia [15]. The topic representation is typically based on sets of keywords or phrases. In probabilistic topic modeling, the topics are represented as a probability distribution over several words. In other approaches, topics are found considering the highly temporal nature of posts. Some other methods utilize similarity measures among posts to identify topics. Among the most recent research works in [16] Hoang et al. proposed a method for simultaneously modeling background topics and users’ topical interest in microblogging data. The approach is based on the realm, which describes a topical user community: users within a realm may not have many social ties among them, but they share some common background interest. In general, a user can belong to multiple realms. De Maio et al. [17] extracted temporal patterns revealing the evolution of concepts along the time from a social media data stream. The extraction process is based on the extended temporal concept analysis and description logic, to reason on semantically represented tweets streams. A microblog summarization algorithm has been defined in [18], filtering the concepts organized by a time aware knowledge extraction method in a time-dependent hierarchy.

The purpose of topic detection in microblogs is to generate term clusters from a large number of tweets. Most topic detection algorithms are based on clustering algorithms. Although traditional text clustering is quite mature, the topic detection method for microblog should focus more attention on temporal aspects. Furthermore, strictly connected to topic detection are the term cloud visualization models. In this section, we highlight the most relevant works in both aspects.

Weng and Lee in [19] present an approach to discover events in Twitter streams, i.e., wavelet analysis or EDCoW. In their approach, the frequency of each word is sampled over time, and a wavelet analysis is applied to this time series. Subsequently, the bursty energy of each word is calculated by using autocorrelation. Then, the cross-correlation between each pair of bursty words is calculated. Finally, a graph is built from the cross-correlation, and relevant events are mined with graph-partitioning techniques. Xie et al. [6] proposed TopicSketch for performing real-time detection of events in Twitter. TopicSketch computes in real-time the second order derivative of three quantities: (i) the total Twitter stream; (ii) each word in the stream; (iii) each pair of words in the stream. Then, the set of inhomogeneous processes of topics is modeled as a Poisson process and, by solving an optimization problem, the distribution of words over a set of bursty topics is estimated. In both [19] and [6], the event is characterized as a bursty topic, which is a trivial pattern in microblogging activities: something happens and people suddenly start writing about it. In contrast to [6,19], our approach computes the similarity between time series without relying on the burst as a special pattern. Thus, the similarity based on stigmergy is more general and flexible. Other approaches are based on a variety of patterns [10,20,21]. Yang and Leskovec [20] developed the K-Spectral Centroid (K-SC) clustering, which is a modified version of classical K-means for time series clustering. K-SC uses an ad-hoc distance metric between time series, which takes in consideration the translation and the shifting to match two time series. More specifically, the authors use long-sized time frames for their analysis, up to 128 h (with one hour as time unit) to detect six common temporal patterns on news and posts. Similarly, Lehmann et al. [21] study the daily evolution of hashtags popularity over multiple days, considering one hour as a time unit. They identify four different categories of temporal trends, depending on the concentration around the event: before and during, during and after, symmetrically around the event, and only during the event. Stilo and Velardi detect and track events in a stream of tweets by using the Symbolic Aggregate approXimation (SAX) [10]. The SAX approach consists of three steps: first the word streams in a temporal window are reduced to a string of symbols; then a regular expression is learned to identify strings representing patterns of collective attention; finally, complete linkage hierarchical clustering is used to group tokens with similar strings occurring in the same window. The authors evaluate the approach discovering event in a one-year stream (with one day as time unit) and manually assessing the events on Google. In particular, five patterns of [10] correspond to six patterns of [20]. This emphasizes a further evidence of the variability of temporal patterns in microblogging. However, while [10,20,21] focus on a coarse temporal scale (hour or day), established at the macro (i.e., application) level, our approach adopt a fine temporal scale (minute) to detect the micro patterns occurring in social discussions, carrying out the analysis of the macro-dynamics without adopting specific patterns at design time.

Xu et al. [7] studied the competition among topics through information diffusion on social media, as well as the impact of opinion leaders on competition. They developed a system with three views: (i) timeline visualization with an integration of ThemeRiver [22] and storyline visualization to visualize the competition; (ii) radial visualizations of word clouds to summarize the relevant tweets; (iii) a detailed view to list all relevant tweets. The system was used to illustrate the competition among six major topics during the 2012 United States presidential election on Twitter. Here, in contrast to our approach, there is no integration of the temporal information in the word cloud which is used as a mean of summarization of the content of the tweet. In [1] Lee developed a cloud visualization approach called SparkClouds, which adds a sparkline (i.e., a simplified line graph) to each tag in the cloud to explicitly show changes in term usage over time. Though useful improvements to term clouds, it does not show term relations and thus it is not able to visualize time-varying co-occurrences. Lohmann et al. extends the term clouds by an interactive visualization technique referred to as time-varying co-occurrence highlighting [2]. It combines colored histograms with visual highlighting of co-occurrences, thus allowing for a time-dependent analysis of term relations. Cui et al. in [3] presented TextFlow, which implies three methods of visualization to describe the evolution of a topic over time: (i) the topic flow view, which shows the evolution of the topics over time; it highlights the main topics and the merging and splitting between topics; (ii) the timeline view, which places document relevant to a selected topic in temporal order; (iii) the word cloud view, which shows the word in different font sizes. In contrast to our approach, the word cloud has no temporal or relational information. Other approaches focus the analysis on the user interest, such as ThemeCrowds [4], which displays groups of Twitter users based on the topics they discuss and track their progression over time. Twitter users are clustered hierarchically and then the discussed topic is visualized in multiple word clouds taken at different interval of time. Tang et al. used a term cloud to represent the interest of the social media users [5]. They presented an SVM classifier to rank the interest of the users, using the user keywords as a score. The score is applied to the font size of the keyword in the term cloud. Raghavan et al. develop a probabilistic model, based on a coupled Hidden Markov Model for users’ temporal activity in social networks. The approach explicitly incorporates the social effects of influence from the activity of a user’s neighbors. It has better explanatory and predictive performance than similar approaches in the literature [23]. Liang et al. observed that (i) the rumor publishers’ behaviors may diverge from normal users; and (ii) a rumor post may have different responses than a normal post. Based on this distinction, they proposed an ad hoc feature and trained five classifiers (logistic regression, SVM with RBF kernel function, Naive Bayes, decision tree, and K-NN). Results show that the performance of rumor classifier using users’ behavior features is better than that based on a conventional approach [24]. In contrast, in our approach we avoid the explicit modeling of the user interest. In this paper, we adopt a new modeling perspective, which can be achieved by considering an emergent design approach [25]. With an emergent approach, the focus is on the low-level processing: social data are augmented with structure and behavior, locally encapsulated by autonomous subsystems, which allow an aggregated perception in the environment. Emergent paradigms are based on the principle of the self-organization of the data, which means that a functional structure appears and stays spontaneous at runtime when local dynamism occurs.

## 3. Formal Approach and Time-Series Dissimilarity

**Figure 3:**
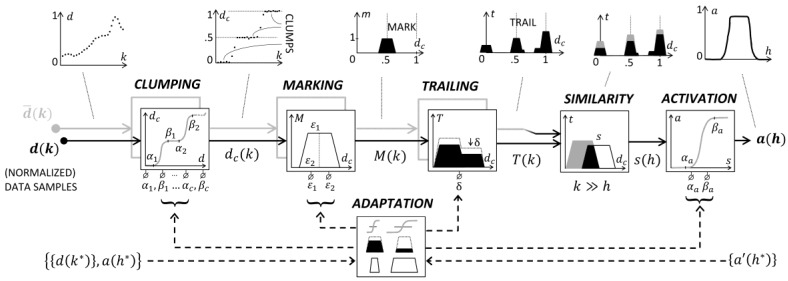
Structure of a Stigmergic Receptive Field.

This section formalizes the proposed approach. In the first part, the input-output information flows with respect to the overview of Figure 3 are defined: term extraction, temporal dynamics construction, and relational term cloud representation. In the remainder, the section focuses on the time-series dissimilarity, represented in Figure 3 as the subsystem between temporal dynamics and dissimilarity matrix. The internal topology of such subsystem is based on a multilayered network of adaptive units. Each unit is structured in terms of two input/output interfaces and three computational processes called marking, trailing and similarity. The unit is adaptive because it is parameterized by an optimization process finding near-optimal interfacing/processing according to similarity samples. Specifically, in the first layer, the units are adapted to find the similarity between the input time series and some predefined social sensing pattern. In the second layer, the unit is adapted to find the similarity between series of patterns detected in the first layer.

More formally, given a stream of tweets E≡{e} related to a specific event, let us consider for each *e* the set of contained terms $\left\{term_{i}\right\}$. A score is assigned to each term: $s(term_{i},e)$ = {1 if $term_{i}\in e$, 0 otherwise}. The aggregated score of $\left\{term_{i}\right\}$ in the stream *E* is $s(term_{i},E)=\sum \;s(term_{i},e):e\in E$.

Then the actual input of the system is represented by the time series related to a term and generated considering the discrete time 0<k≤K of consecutive, non-overlapping temporal slots of fixed length. The element d(k) of the time series is the score $s(term_{i},E(k))$, where E(k)⊂E is the sub-stream of tweets published in slot *k*.

The overall output of the system is a relational term cloud, where the font size of $term_{i}$ is linearly proportional to $s(term_{i},E)$ scaled to the range [*minFontSize*, *maxFontSize*] defined by the user. The relational term cloud is built by means of term similarity obtained from the similarity between the related time series. 

To calculate the similarity between two time series, we propose a connectionist architecture which relies on bio-inspired scalar and temporal aggregation mechanism, in the following called stigmergy [11,26,27]. The aggregation result is called trail, which is a short-term and short-size memory, appearing and staying spontaneously at runtime when local dynamism occurs. In the proposed architecture the trailing process is managed by units called Stigmergic Receptive Fields (SRFs). SRFs are parametrically adapted to contextual behavior by means of the DE algorithm. The concept of receptive field is the most prominent and ubiquitous computational mechanism employed by biological information processing systems [28]. In our approach, it relates to a general purpose local model (archetype) that detects a micro-behavior of the time series. An example of micro-behavior is rising topic, which means that a term shows a sudden increase of occurrences over time. Since micro-behaviors are not related to a specific event, any receptive field can be reused for a broad class of events. Thus, the use of SRFs can be proposed as a more general and effective way of designing micro-pattern detection. Moreover, SRF can be used in a multilayered architecture, thus providing further levels of processing to realize a macro analysis. 

Basically, the final purpose of an SRF is to provide a degree of similarity between a current micro-behavior, represented by a segment of time series, and an archetype micro-behavior, referring to a pure form time series which embodies a behavioral class. 

Figure 3 shows the structure of a single SRF where $\bar{d}(k)$ and d(k) denote the values of two time series, archetype, and input segments respectively, at time *k*. The first three processing modules of the SRF are the same for the archetype and the input segment. To avoid redundancy in the figure, these modules for the archetype, and their respective inputs, are represented as gray shadow of the corresponding ones.

Before processing, a min-max normalization of the original time series is considered. It is a linear mapping of the data in the interval [0, 1], i.e., *d*(*k*) = [*d*(*k*) − *d*_MIN_]/[*d*_MAX_ − *d*_MIN_], where the bounds *d*_MIN_ and *d*_MAX_ are estimated in an observation time window. To assure samples are positioned in-between 0 and 1, the results are clipped to [0, 1].

The normalized data samples are processed by the module clumping, in which samples of a particular range group close to one another. Clumping is a kind of soft discretization of the continuous-valued samples to a set of levels. Second, with the marking processes each data sample produces a corresponding mark, represented as a trapezoid. Third, with the trailing process, the accumulation and the evaporation of the marks over time create the trail structure. Fourth, the similarity compares the current and the archetype trails. Fifth, the activation increases/decreases the rate of similarity potential firing in the cell. Here, the term “activation” is borrowed from neural sciences: it inhibits low intensity signals while boosts signals reaching a certain level to enable the next layer of processing [29].

Each SRF should be properly parameterized to enable an effective aggregation of samples and output activation. For example, short-life marks evaporate too fast, preventing aggregation and pattern reinforcement, whereas long-life marks cause early activation. This adaptation is part of the novelty of the undertaken study [25,30]. The adaptation module uses the DE algorithm to adapt the parameters of the SRF with respect to the fitness function. The fitness function is the Mean Squared Error (MSE) between the output the SRF provided for a certain input and the desired output provided in the tuning set for the same input. A better explanation of the parameters’ adaptation process can be found in Appendix A. In Figure 3, the tuning set is denoted by asterisks: it is a sequence of (*input*, *desired output*) pairs, on the left side, together with a corresponding sequence of *actual output* values, on the right side. In a fitting solution, the desired and actual output values corresponding to the same input are made very close to each other.

**Figure 4:**
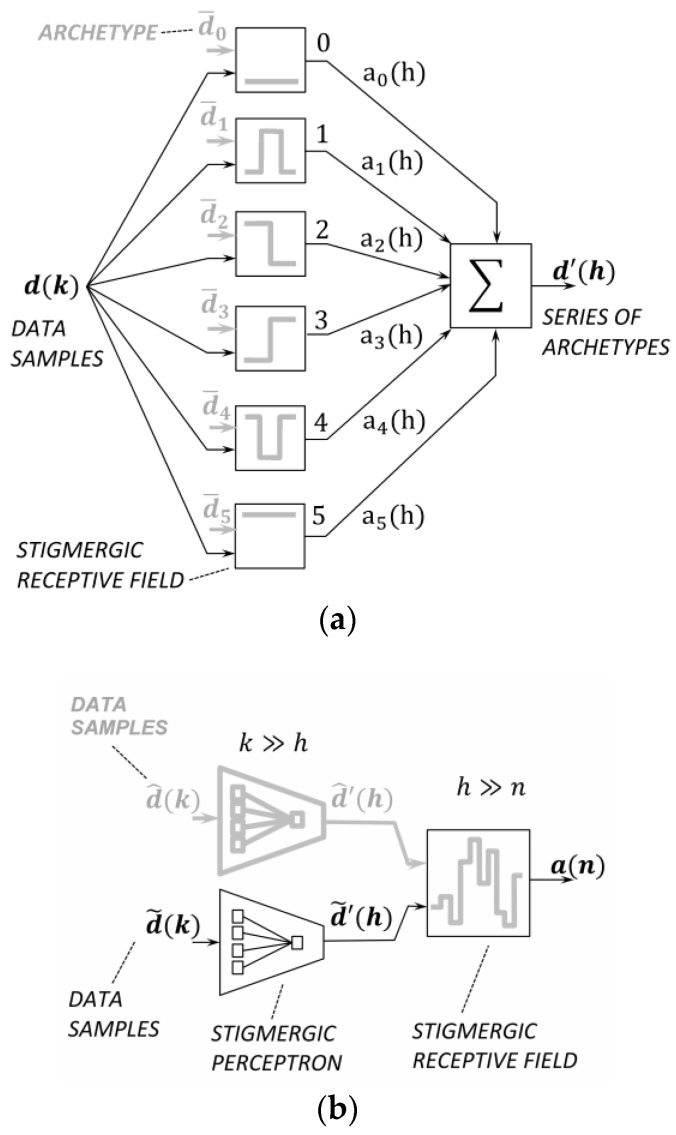
(**a**) Topology of a stigmergic perceptron; (**b**) Topology of a multilayer architecture of SRF.

Figure 4a shows the topology of a stigmergic perceptron. The stigmergic perceptron performs a linear combination of SRF results parameterized for achieving some desired mapping [28].

More specifically, Figure 4a shows six SRFs. Each SRF is associated to a class in a totally ordered set. Indeed, an ordering relation between the archetypes can be defined considering the barycenters of the related trails. Thus, the output of each SRF is a prefixed natural number in the interval [0, 5]. In the output layer, the average of the natural numbers of each SRF is weighted by the related activation. As a result, a linear combination of the SRFs outputs in the interval [0, 5] is provided.

Figure 4b shows the topology of a multilayer architecture: in the first layer, a scalar-temporal micro clustering based on the similarity detection to archetypal patterns is provided. A higher-level time series is then generated through each stigmergic perceptron and analyzed by another SRF. The adaptation of this SRF is based on samples provided by an application domain expert. This layer carries out a macro-level similarity between two time series. The next subsections formalize each module of the architecture of Figure 4b.

### 3.1. The Input/Output Interfaces of SRF

**Figure 5:**
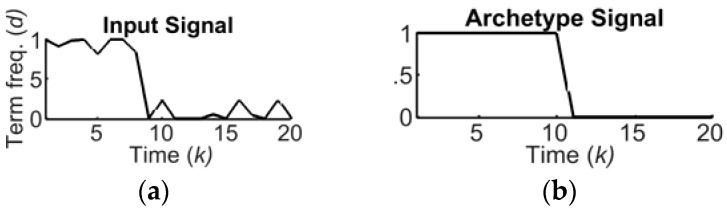
(**a**) Example of input signal for an SRF; (**b**) Corresponding archetype signal.

Micro-patterns of changes over time [31] are illustrated in Figure 4a: *dead topic (0), cold topic (1), falling topic (2), rising topic (3), trending topic (4)*, and *hot topic (5)*. Figure 5 shows an example of input data for an SRF and an archetype, i.e., the pattern for a *falling topic*. Each SRF is assigned a different micro-pattern.

The similarity value, 0≤s(h)≤1, is close to 1 if the signal is very similar to the archetype. Since a single output similarity sample $d^{\prime }(h)$ is provided with many input data samples {d(k)}, it follows that k≫h. Figure 5 shows that *k* = 20 data samples, which can provide one (*h* = 1) similarity sample.

**Figure 6:**
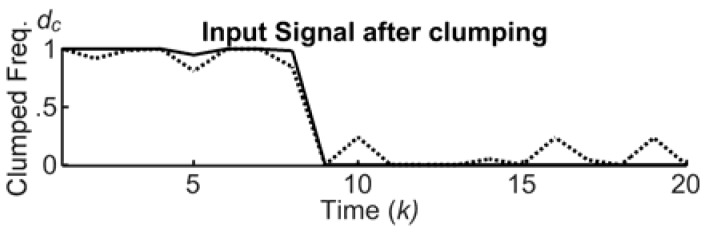
The clumping process, with two levels, $\alpha_{1}=0.25$ and $\beta_{1}=0.75$.

Figure 6 shows the clumping process. Here, the input and the output signals are represented with dashed and solid lines, respectively. It is evident that the clumping reduces the ripples and allows for a better distinction of the critical phenomena during unfolding events, with a better detection of the progressing levels. The clumping is generated by multiple s-shape functions, endowed with 2⋅c parameters $\left(\alpha_{1},\beta_{1},\ldots ,\alpha_{C},\beta_{C})\in [0,1\right]$. More formally: (a) input values in the interval $\left[0,\alpha_{1}\right]$ are mapped to level 0; input values in the interval $\left[\beta_{i},\alpha_{i+1}\right]$ are mapped to level i/c, and so on; input values in the interval $\left[\beta_{C},1\right]$ are mapped to level 1. Continuous values in the interval $\left[\alpha_{i},\beta_{i}\right]$ are softly/hardly transferred into discrete counterparts or levels, depending on the distance between $\alpha_{i}$ and $\beta_{i}$. All archetypes shown in Figure 3 use two levels, i.e., *c* = 1.

In essence, at the output interface the activation process is always generated on two levels by a single s-shape function. Thus, the activation is set via a pair of parameters $\left(\alpha_{a},\beta_{a}\right)$, as represented in Figure 3.

### 3.2. The Marking Process

**Figure 7:**
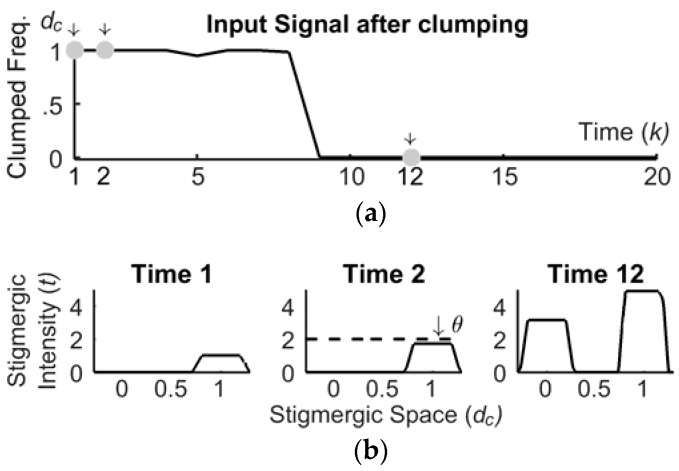
The marking and the trailing processes: (**a**) time domain; (**b**) stigmergy domain.

The marking takes a clumped sample, $d_{c}(k)$ in Figure 3, as an input, and releases a mark M(k) to the trailing. A mark has three structural attributes: the center position $d_{c}(k)$, the intensity *I*, and the extensions of the upper/lower bases, i.e., *ε*_1_/*ε*_2_. Figure 7 shows an input signal after clumping, and the release of three trapezoidal marks at the time ticks 0, 2, and 12, with intensity *I* = 1, upper and lower bases $\varepsilon_{1}=0.3$ and $\varepsilon_{2}=1.5\cdot \varepsilon_{1}$, in a time window of 20 total ticks. More precisely, the sample $d_{c}(1)=1$ generates the first mark centered on 1.

### 3.3. The Trailing Process

After each tick, the mark intensity decreases by a value θ, 0<θ<1, called evaporation rate. Evaporation leads to progressive disappearance of the mark. However, subsequent marks can reinforce a previous mark when overlapping. Figure 7 shows, at time 2, the release of a second mark on the trail, T(k), which is made by the first mark evaporated by a factor θ=0.25. For this reason, the new trail is lower than 2. Finally, Figure 7 shows, at time 12, the evolution over time of the trail. Here, the right trapezoid is higher than the left one, because more marks were recently released on 1 with respect to 0. This example shows that the stigmergic space acts as a scalar and temporal memory of the time series.

### 3.4. The Similarity Process

**Figure 8:**
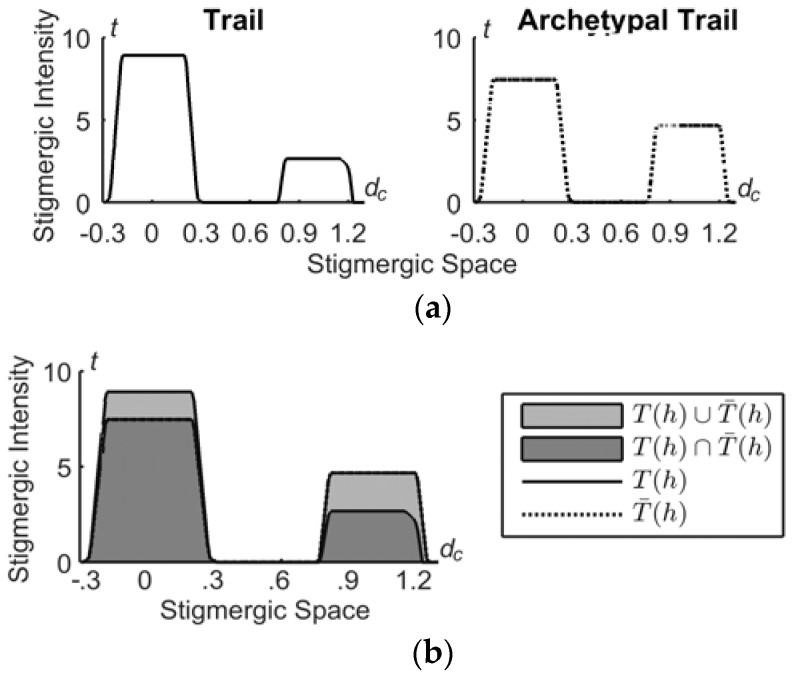
(**a**) Two trails and (**b**) their similarity.

Similarity takes two trails as inputs, i.e., the archetype trail, $\bar{T}(h)$, and T(h), to provide their similarity *s(h)*. As a similarity measure we adopt the Jaccard’s index [32], calculated as follows. Let *A*(*x*) and *B*(*x*) be two trails defined on the variable *x*; the similarity is calculated as |*A*(*x*)∩*B*(*x*)|/|*A*(*x*)∪*B*(*x*)| where *A*(*x*)∩*B*(*x*) = min[*A*(*x*), *B*(*x*)], *A*(*x*)∪*B*(*x*) = max[*A*(*x*), *B*(*x*)]. Thus, the similarity is maximal, i.e., 1, for identical sets, and decreases progressively to zero with the increase of the non-overlapping portion, as shown in Figure 8.

### 3.5. The Stigmergic Perceptron

**Figure 9:**
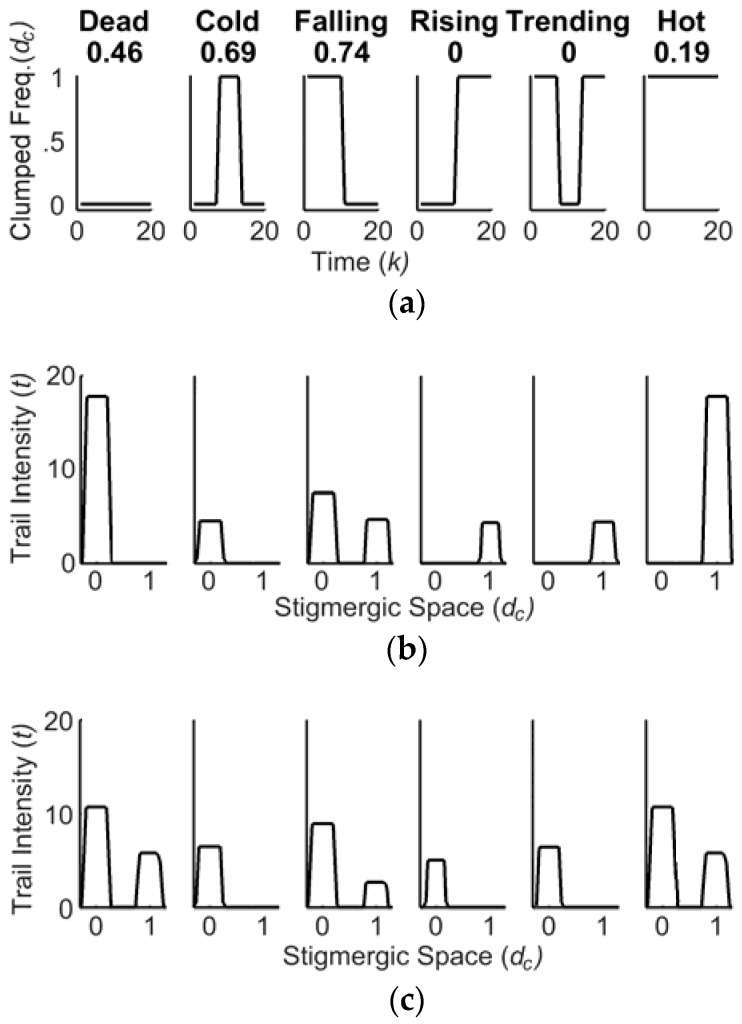
(**a**) Archetypes for social discussion topics, and related similarity between (**b**) archetypes trails and (**c**) an input signal trails.

The Stigmergic Perceptron (SP) compares an input time series fragment d(k) with a collection of archetypes *P*, using SRFs, and returns an information granule $d^{\prime }(h)$ reflecting the activation of some close SRFs. In the stigmergic space each SRF is associated to a prefixed integer number in the interval [0, P − 1]. Figure 9a,b show the considered archetypes and the related trails, respectively.

Here, the trails show a progressive shift of the barycenter from 0 to 1, which clearly is an ordering relation.

The output information granule is taken as the average of the SRFs natural numbers, weighted by their activation levels:

(1) $$d^{\prime }(h)=\sum_{1}^{p}p\cdot a_{p}(h)/\sum_{1}^{p}a_{p}(h)$$

where $a_{p}(h)\in [0,1]$ is the output of the *p*^th^ SRF.

(2) $$s(h)=\frac{T(h)\cap \bar{T}(h)}{T(h)\cup \bar{T}(h)}$$

Figure 9c shows in solid line the trails of the pilot signal in the six SRFs. Figure 9a top shows also the similarity value between the input trails and the corresponding archetype trails. In the case of Figure 9a, the overall output for the pilot time window is ${\tilde{d}}^{\prime }(h)=2$, which identifies the falling topic behavior.

### 3.6. The Second Layer SRF

The resulting information granule provided by a stigmergic perceptron represents a sample of a second level time series. In the proposed architecture, at the second (macro) level, two time series are compared using another SRF, as represented in Figure 4b. The overall comparison process uses sliding time windows that overlap for the half of their length, to assure a smooth output time series. The resulting activation uses again the Jaccard’s index.

In the next section, the output activation value a(n) is used as an input of a relational clustering algorithm. For clarity and correspondence with distance functions, the value 1−a(n) is actually provided. In other terms, the complementary value, called dissimilarity value, is calculated in place of similarity. The dissimilarity value is close to 0 (1) if the two time series show the same (different) temporal dynamics. Finally, a D×D matrix between the *D* time series related to terms can be generated.

## 4. Relational Clustering and Relational Term Cloud

In the first part of this section, the relational clustering subsystem is formalized. With respect to the overview of Figure 3, this subsystem is between the dissimilarity matrix and the relational term cloud. The dissimilarity matrix represents each term as an array of similarity values with the other terms. In Figure 3, the degree of dissimilarity is painted as a grey level from 0 (black) to 1 (white). A brief analysis of this structure reveals some internal blocks of terms with high mutual similarity. The purpose of the relational clustering is to assign similar terms to the same cluster and dissimilar terms to different clusters. As a final output, the relational term cloud represents each cluster with a different color. In the remainder, this section includes other details on the relational term cloud.

More formally, given a D×D dissimilarity matrix between terms, the purpose of the clustering process is to visualize the terms on a two-dimensional space, as a relational term cloud (RTC).

In an RTC, terms are grouped according to their scalar-temporal usage, thus generating different clusters each characterizing a social discussion topic.

To produce a two-dimensional representation of the matrix, we adopt a Self-Organizing Map (SOM), developed with the use of unsupervised learning [33]. A SOM applies competitive learning on the matrix to preserve the topological properties of the matrix itself. This makes SOM effective to produce a two-dimensional view of high-dimensional data.

The SOM exhibits a single bi-dimensional layer of $n_{1}\times n_{2}$ neurons, each made of a *D*-dimensional array of weights, whose training causes different part of the network to respond similarly to similar input patterns. This behavior is partly inspired by how visual, auditory, and other sensory information is handled in separate parts of the cerebral cortex of the brain and matches with the concept of receptive field. More specifically, in the training of the network the weights of the neurons are initialized to random values. Iteratively (i) each row of the matrix is fed to the network; (ii) its Euclidean distance to all neurons is computed; (iii) the weights of the most matching neurons are then adjusted to be closer to the input.

A common feature of SOM is to reduce the dimensionality of data [34]. It means that information about distance and angle is lost in the training process but proximity relationship between neighbor neurons is preserved: neurons which are close one to another in the input space should be close in the SOM space. Since each neuron becomes a representative of a discussion topic as a group of patterns in the input data set, discussion topics can be represented on a 2D-space, exploiting the Euclidean distances between neurons. For this purpose, we adopt a Force-directed graph drawing algorithm [35], which positions the nodes of a graph in two-dimensional space. In essence, the algorithm assigns forces among the set of edges and the set of nodes, based on their relative positions, and then uses these forces either to simulate the motion of the edges and nodes or to minimize their energy.

As a result, each discussion topic is represented in a different color, and the relative position between different discussion topics reflects the corresponding proximity between nodes of the SOM. More specifically, each cluster of terms is then represented as a term cloud whose barycenter is placed on the position assigned by a force-directed graph algorithm [36]. In particular, the score of each term $s(term_{i},E)$ determines the font size of *i*-th term. The most used terms appear more visible in the picture, and discussion topics which are scalarly and temporally related appear closer.

**Figure 10:**
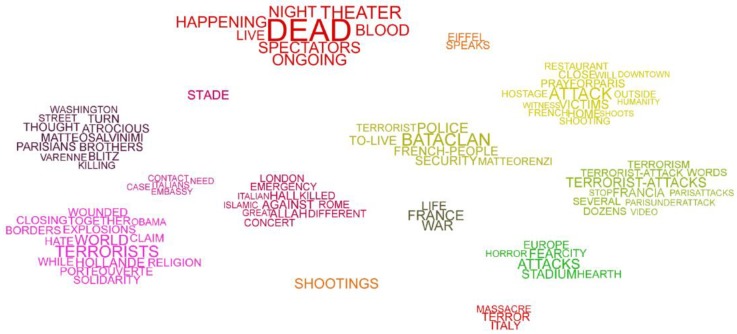
The relational term cloud with some relevant social discussion topics.

Figure 10 shows the relational term cloud generated from the overall process. The major terms are located on different topics (clusters), i.e., *dead*, *attack*, *Bataclan*, *terrorists*, and *shootings*. On the bottom-left side, there is a topic regarding the political speech and the emerging reactions. Here, the term *terrorists* was one of the most used overnight, especially in conjunction with the speech of Barack *Obama* and Francois *Hollande*, which began the speech with *solidarity* towards the *brothers* and *Parisians*. Indeed, the last two terms belong to the neighboring cluster. Another political sub-topic raised by the community was the management of *borders*. At the same time, an appeal to *Italians* in Paris that may *need*, was spread retweeting details of the Italian *Embassy*. The relevance of this topic, represented by a small neighboring cluster, is caused by the filter by Italian language which applied to the posts stream.

The next cluster on the right is focused on people *killed* at the *concert*, *hall*, and the strong connection with *Islamic*, *religion*, which raises the *emergency* level for *Rome* and *London*. Moreover, the community expressed disagreement using the expression “*Allah* is not *great*”. On the top-middle, the most important term is *dead*, widely used overnight when reporting the *ongoing* events, producing *blood* at the *theater* and at the *Stade*. On the right side, there are different discussions topics, on details about the story. The terms *attack*, *terrorist-attacks*, *Bataclan*, *victims* are the most prominent and distinguish different aspects. More specifically, on the top-right there is the *shooting* at the *downtown*, at the *restaurant*, with the presence of *hostage* and *victims*. It is worth noting how the terms *shooting* and *shootings* are quite distant in the cloud. The reason is that the term *shooting* (singular) has been mainly used to report the attacks at the *restaurant*, whereas the term *shootings* (plural) has been largely used for all the overnight events. Indeed, the latter is represented as an individual cluster. At the bottom-right, the terms related to *terrorism* and *terrorist-attack(s)* are grouped together. Nearby, the *horror* and the *fear* emerge because of the attack at the *heart* of a *city* of the *Europe*. Finally, at the bottom, a well-defined discussion topic on the *terror* of a possible *massacre* in *Italy*.

Each cluster represents an entire or part of a discussion topic. The purpose of this paper is to present a technique to extract the structure of relationships between terms. Once the terms are clustered, the system assigns a numeric value for each clustered group. An important future development is to assign meaningful labels to each cluster, thus providing cluster names. For example, some labels that may summarize the topics of Figure 10 are “attack story”, “debate on terrorism”, “speech of political leaders”. This task can be handled by a domain expert, or via computational linguistics techniques, which are out of the scope of this research.

## 5. Experiments and Results

The proposed architecture has been tested with different types of socially relevant events, ranging from musical/sport competition to political elections, from weather emergency to terrorist attacks. For the sake of significance, in this section we focus on the record highlighting the most important properties of the approach: a dataset of 188,607 Twitter posts collected during the terrorist attacks in Paris on 13 November 2015, between the 9 p.m. on the 13 and the 2 a.m. on the 14 (BBC News, 9 December 2015, *Paris attacks: What happened on the night*, http://www.bbc.com/news/world-europe-34818994).

Several challenges are related to data capturing and filtering of social media data. The challenge related to data capturing lies in gathering, among the sheer amount of social media messages, the most complete and specific set of messages for the detection of a given type of social event. Moreover, since not all the collected messages are actually related to an unfolding event, there is the need of a data filtering step to further reduce the noise among collected messages and retain only the relevant ones. Moreover, techniques are needed to analyze relevant messages and infer the occurrence of an event. In this section, we briefly summarize the most important steps of the process and of the supporting architecture. The reader is referred to [37,38].

From the overall collection of tweets related to the event, the first step is to remove the stop words and the historical baseline. The stop words are the most common words universally used in a language, with relevant statistics but without trends in the occurrence of an event, such as *the*, *is*, *at*, *which*, and so on. The historical baseline is a set of sources and related terms generally related to the type of the event rather than to the specific occurrence, such as the posts of press agencies, journalists, and bloggers, since the purpose is to identify terms, which make deviations from the historical baseline [37]. To carry out an accurate text preprocessing, the analysis was made considering only posts in the mother language of the data analyst (i.e., Italian). For the sake of readability, in each figure the terms have been translated to English.

Once the above process has been completed, the top 150 words ranked by frequency have been selected. For each word, the corresponding time series is generated over the collection of tweets, using temporal slots of 1 min. The resulting set *D* of time series has been labeled, in an annotation campaign involving externally enrolled annotators [37]. The task assigned to the annotators was the following: given a time series, assign it to a group of similar time series, considering *L* groups. The groups are initially empty, and then the process is essentially iterative. For each annotator, the process was repeated three times, i.e., for *L* equals to 10, 15, and 20. In case of disagreement between annotators, the membership of a time series to a cluster was decided according to the principle of majority, otherwise the disputed term is discarded. At the end of the annotation process, the collection of time series was divided into *L* clusters, providing |*D*| = 102 time series and their corresponding cluster labels *L* * (*i*).

### 5.1. Clustering Performance

To assess the performance of the clustering we use the B-cubed cluster scoring, which decomposes the evaluation of the clusters estimating the precision and the recall of each item of the dataset [39]. More formally, for a given item *i*, let us consider *L* * (*i*) and *L*(*i*), i.e., the actual label and the cluster label, respectively. The correctness of the relation between two items *i*, *j* reflects the situation that two items are correctly clustered together and they have the same label:

(3) $$Correctness(i,j)=\left\{ \begin{array}{ll} 1 & \mathrm{if}\;L(i)=L(j)\;\&L*(i)=L*(j)\\ 0, & \mathrm{otherwise} \end{array}\right.$$

The B-cubed Precision of an item is the proportion of items in its cluster that have the same item’s label (including itself). The overall B-cubed Precision is the average precision of all items. The B-cubed Recall definition is analogous to Precision, replacing the word “cluster” with the word “label”:

(4) $$P=avg_{i,j}{\left[Correctness(i,j)\right]|}_{L(i)=L(j)}$$

(5) $$R=avg_{i,j}{\left[Correctness(i,j)\right]|}_{L*(i)=L*(j)}$$

Finally, the B-cubed *F-measure* assesses the performance of the cluster:

(6) $$F_{1}(R,P)=2\cdot R\cdot P/(P+R)$$

### 5.2. Numerical Performance of the Clustering Process

In this section, we compare the result of the clustering of time series by using three different distance measures: (i) our similarity measure based on the Multilayer SRF architecture (M-SRF for short), the Dynamic Time Warping (DTW) distance [13,14], and the Euclidean distance (E). We also test the benefits of min-max normalization of input data on the last two techniques, N-DTW and N-E for short, respectively. Table 1 summarizes the result for different numbers of labels/clusters. A first aspect to highlight is that E and DTW distances exhibit similar performance. This may be ascribed to the fact that the time series do not have temporal drift nor temporal scaling [11]. Thus, the robustness to temporal shift, which is a distinctive feature of DTW, is not exploited. A second aspect is that normalization improves the performance for both the ED and the DTW. The most important result is that the M-SRF outperforms the other measures, thus confirming the effectiveness of our approach. In particular, the M-SRF achieves the best performance using a SOM with 5 × 3 neurons (i.e., 15 clusters), whereas the DTW and E achieve the best performance with 5 × 4 neurons (20 clusters). In general, we remark that the performance is not the unique criterion to consider: interpretability of the cloud is also important. Regarding interpretability, 20 clusters can be considered as an upper bound.

### 5.3. Considerations on Efficiency and Scalability of the Proposed Approach

To better highlight the efficiency and scalability of the proposed approach on real microblog environment, with large volumes of real time data and complex topics, we have experimented it on several global events of different nature: emergencies, culture, games, sport, and politics. In the following, we summarize some considerations on one of the most discussed events of 2016, i.e., the election of the president of the United States, a very complex and long campaign. In particular, the last public debate was the most followed: Donald Trump accumulated more than 1.2 million mentions on Twitter while Hillary Clinton, received a little over 809,000 mentions [40,41]. The debate was scheduled for Wednesday, 19 October 2016 at 21:00 EDT, i.e., Thursday, 20 October 2016 at 03:00 CEST, for one and half hour. In the post history, they were captured 2,052,790 tweets between Wednesday, 19 October 2016 at 22:50 CEST and Thursday, 20 October 2016 at 11:35 CEST. However, the significant dynamics emerged on Thursday, 20 October 2016 between 02:00 and 05:30 CEST, concentrated on overall 602,532 tweets. The overall process is fed by the post capturing. To guarantee the robustness and the reliability of the post capturing, in [33] we have described a detailed system implementation, supporting mechanisms that manage rate-limit and generic connection problems.

In the following, a representative setting is summarized. The interested reader is referred to Appendix A for further details on the parameterization. Filtering stop words, bad words, and other generally common terms, the term extraction activity has selected 106 major terms. As an output, a new term cloud is generated by the system every 30–60 min time window. By using a workstation with 40 cores, Intel Xeon CPU 2.40 Ghz, 128 GB RAM, Ubuntu Server 18 LTS Operating System, the proposed approach can effectively manage a delivery rate of about 30–60 min. More specifically, the delivery rate can be controlled via several strategies, summarized in what follows. First, the most of the configuration/parameterization steps can be carried out one time and reused for broad classes of events. Indeed, the term extraction process relies on linguistic filters based on wordlists that can be tuned one time and reused for many events related to politic debates. Furthermore, in the time-series dissimilarity, the adaptation process of the first layer is based on micro-archetypes that are very common in social sensing. On the other hand, in the second layer the range of each parameter can be highly constrained to sensibly reduce the adaptation time. Finally, time series of each term can be incrementally created and immediately used when necessary.

## 6. Conclusions

In this paper, we have presented a novel approach to identifying event-specific social discussion topics from a stream of posts in microblogging. The approach is based on deriving a scalar and temporal similarity measure between term occurrences, and generating a relational term cloud, i.e., a cloud whose term positions are related to the similarity measure.

To derive the similarity measure from data, we have developed a novel multi-layer architecture, based on the concept of Stigmergic Receptive Field (SRF). The stigmergy allows the self-aggregation of samples in a time series, thus generating a stigmergic trail, which represents a short-time scalar and temporal behavior of the series. In the stigmergic space, the similarity compares the current series with a reference series. The recognition of the combined behavior of multiple SRF models is made by using a stigmergic perceptron. The similarity is used to guide a Self-Organizing Map, which carries out a clustering of the terms.

Experimental studies completed for real-world data show that results are promising and consistent with human analysis, and that the M-SRF similarity outperforms both the DTW and the Euclidean distances.

## Figures and Tables

**Table 1:** Performance of the M-SRF and the DTW distances.

|L|	F-Measure ± 95% Confidence Interval				
	DTW	N-DTW	E	N-E	M-SRF
10	0.299 ± 0.005	0.355 ± 0.008	0.297 ± 0.007	0.360 ± 0.006	0.383 ± 0.009
15	0.301 ± 0.006	0.341 ± 0.005	0.309 ± 0.007	0.334 ± 0.008	0.423 ± 0.010
20	0.339 ± 0.003	0.389 ± 0.003	0.344 ± 0.004	0.386 ± 0.004	0.410 ± 0.004

**Figure A1:**
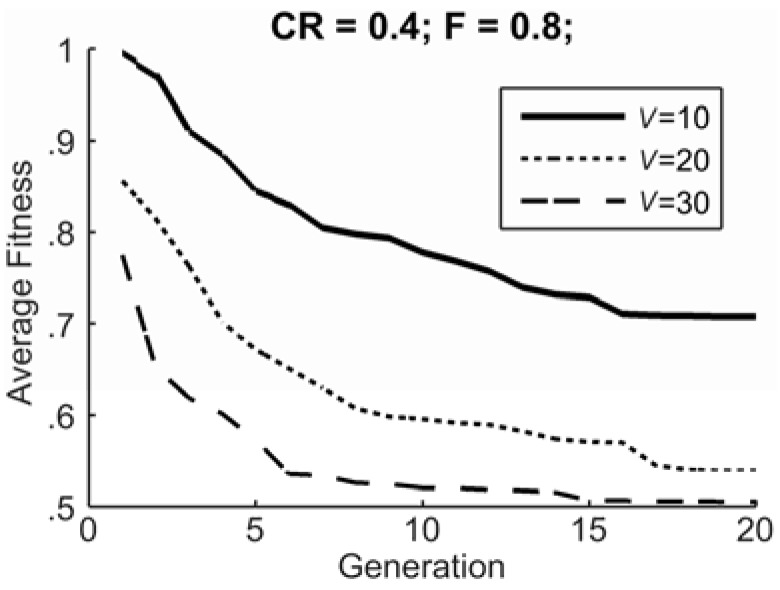
The average fitness of the population against the generation.

## Appendix A. Parameters Adaptation

The introduced multilayer architecture requires a proper parameterization to generate a suitable similarity measure. More specifically, the temporal parameters τ and *K* regulate the granularity of the analysis. They depend on the span (first and last timestamp), the volume of the stream of tweets *E*, and the dynamics of the events. The parameters of the SRF, i.e., $\alpha_{c}$, $\beta_{c}$, *ε* (setting *ε* = *ε*_1_, and fixing the ratio *ε*_1_/*ε*_2_ to a constant value), δ, $\alpha_{a}$, $\beta_{a}$, depend on the parameter *K* and the shape of the archetypal signals. At the first layer, since each SRF is specialized to recognize a specific scalar-temporal pattern, for each SRF a different adaptation process is carried out by the DE algorithm. At the second layer, the adaptation of the SRF is based on a set of similarity measures between terms established by a group of analysts of the event.

### A.1. The Differential Evolution Algorithm

In the DE algorithm, a solution is represented by a real *v*-dimensional vector, where *v* is the number of parameters to tune. Here, this vector has 6 elements *v* = ($\alpha_{c}$, $\beta_{c}$, *ε*, θ, $\alpha_{a}$, $\beta_{a}$). The fitness function is specified as the MSE between the output of similarity calculated by the module and the corresponding similarity provided for the related tuning inputs:

(A1) $$f(Z)=\sum_{h=1}^{\left|Z\right|}{\left(a(h*)-a^{\prime }(h*)\right)}^{2}/|Z|$$

The objective of the optimization is to minimize the fitness function. In the tuning set, the target values *a*(*h**) provided for each SRF are 1 (similar) and 0 (dissimilar). At each generation of the DE, a population *V_g_* = {*v*_1,*g*_,…, *v_|V|_*_,*g*_} of candidate solutions is generated, where *g* is the current generation. In the literature, different ranges of population are suggested [42]. Population size can vary from a minimum of 2⋅|V| to a maximum of 40⋅|V|. A higher size of the population increases the chance to find an optimal solution, but it is more time consuming. At each iteration, and for each member (target) of the population, a mutant vector is first generated by mutation of selected members, and a trial vector is then generated by crossover of mutant and target. Finally, the best fitting member among trial and target replaces the target [11].

The DE algorithm itself can be configured by using three parameters [42]: the population *N*, the differential weight *F*, and the crossover rate *CR*. To avoid premature convergence towards a non-optimal solution, such parameters should be properly tuned. Many strategies of the DE algorithm have been designed, by combining different structure and parameterization of mutation and crossover operators [43]. We adopted the *DE*/*1*/*rand*/*bin* [44] version, which computes the mutant vector as:

(A2) $$v_{m}=v_{r_{1},g}+F\cdot (v_{r_{2},g}-v_{r_{3},g})$$

where $v_{r_{1},g},v_{r_{2},g},v_{r_{3},g}\in V_{g}$ are three randomly selected population members, with $r_{1}\neq r_{2}\neq r_{3}$. The differential weight *F* ∈ [0, 2] controls the amplification of the differential variation. *F* is usually set in the range [0.4, 2] [43]. There are different crossover methods for the generation of a trial vector $v_{b}$. Results show that a good approach is generally based on the binomial crossover [44]. With binomial crossover, a component of the offspring is taken with probability *CR* from the mutant vector, and with probability 1-*CR* from the target vector. A sound value of *CR* is between 0.3 and 0.9 [1].

### A.2. Definition of the Tuning Space

When some domain knowledge is available, the search space of each parameter can be constrained to improve and speed up the convergence of the SRF adaptation. In [11] the authors pointed out that DE can benefit from: (i) the limitation of the space domain of each parameter; (ii) the injection of a solution based on human-expert knowledge. In terms of limitation to the space domain, SRFs at the first layer should handle more scalar and temporal perturbation (e.g., ripple, amplitude scaling, drift, offset translation, and temporal drift [11]), than the SRF at the second layer. Indeed, the first layer is fed with raw samples, whereas the second layer is fed with archetypal patterns. For this reason, with respect to the total search space of each layer (i.e., [0, 1] and [0, 5], respectively) the space domain at the second layer is narrower than at the first one.

More specifically, considering the clumping process, the search space for each SRF at the first and the second layers is $\alpha_{c}\in [0,0.4]$, $\beta_{c}\in [0.6,1]$, and $\alpha_{c}=[0,0.05]$, $\beta_{c}=[0.95,1]$, respectively. Such intervals have been established by means of a preliminary estimation of the noise at the input time series of each SRF. We remark that the input noise in the two levels differs by a factor of about 10. In the activation process, low/high values of both $\alpha_{a}$ and $\beta_{a}$ facilitate/impede the sudden activation of the SRF, whereas different values of them enable a soft and incremental activation. The search space for each SRF at the first and the second layers is $\alpha_{a}\in [0.45,0.75]$, $\beta_{a}\in [0.8,1]$, and $\alpha_{a}\in [0,0.2]$, $\beta_{a}\in [4.8,5]$, respectively. Let us consider the marking processes. For the sake of interpretability of the trails, the ratio *ε*_1_/*ε*_2_ = 2/3 of trapezoids is fixed. Thus, the parameter *ε* = *ε*_1_ regulates the spatial aggregation of the marks and is a means to absorb scalar perturbation. Indeed, if *ε* is close to 0, two consecutive marks cannot interact with each other unless the two samples are identical. If *ε* is close to 1, all marks reinforce each other without distinction between patterns. Thus, the related search space has been set considering the average perturbation and variability in the input signal, at the first and second layer: ε∈[0,0.25] and ε∈[0,3], respectively. Considering the trailing process, the evaporation δ is a mean to absorb the temporal variations between samples controlling the duration of the trail. A low value of δ implies a long duration of the trail, and then it becomes possible to aggregate samples occurring in very different instants of time. In contrast, a high value of δ implies that only subsequent samples can aggregate. At the first layer, the related search space has been set to allow a distinction between the archetypes *cold*, *rising*, and *falling*:δ∈[0.4,0.9]. At the second layer, δ should be sufficiently low to allow long-term similarity between the two time series; these values are taken from the range [0.05, 0.3].

Table A1 shows, for each parameter of a first-layer SRF and for each archetype, the value generated by human expert tuning (HE), and the value generated by DE algorithm. In the case of human tuning, the adaptation process adjusted a parameter per time in the same order of the data processing. In contrast, the DE algorithm investigates solutions with a higher resolution than human expert. More precisely, the human expert explored solutions with resolution 0.1, whereas DE can achieve resolution 0.01, which is very time consuming for humans.

The HE values reported in Table A1 have been found by using the following heuristic rules. We adjust one parameter per time, in the same sequence of the data processing. Let us consider the sets of examples labeled, *dead topic* and *hot topic*. In the clumping process, the intervals $\alpha_{c}\in [0,0.4]$ and $\beta_{c}\in [0.6,1]$ are sufficient to include all the occurrences of a *dead topic* and a *hot topic*, respectively. The values set are the mean values of such intervals, i.e., $\alpha_{c}=0.2$ and $\beta_{c}=0.8$. The same values are set for all the SRFs. In the marking and the trailing process, the mark width *ε* regulates the spatial aggregation of the marks and it is an important means to absorb scalar noise such as amplitude scaling, offset translation, linear drift and temporal drift [11]. The mark evaporation θ is a sort of short memory of the trail. A low (high) value implies a long (short)-term memory. For *Cold*, *Rising*, *Falling*, and *Trending topic*, which are characterized by variation between levels, the intervals θ∈[0.4,0.6] and ε∈[0,0.3] guarantee a good distinguishability between trails. The values set are the mean values of such intervals, i.e., *ε* = 0.1 and θ=0.5. For *Dead topic* and *Hot topic* both parameters can be increased to allow a better distinguishability, i.e., *ε* = 0.2 and θ=0.8. Finally, for the activation $\alpha_{a}$ and $\beta_{a}$: in general, high values of both are required if there are signals with similar pattern otherwise it is advisable to set low the values to facilitate the activation of the SRF.

**Table A1:** The human expert tuning (HE) and the DE adaptation for each first layer SRF.

Archetype		*Dead*	*Cold*	*Rising*	*Falling*	*Trending*	*Hot*
$\alpha_{c}$	HE	0.2	0.2	0.2	0.2	0.2	0.2
	DE	0.23	0.30	0.06	0.4	0.36	0.23
$\beta_{a}$	HE	0.8	0.8	0.8	0.8	0.8	0.8
	DE	0.93	0.70	0.82	0.71	0.74	0.93
ε	HE	0.2	0.1	0.1	0.1	0.1	0.2
	DE	0.06	0.03	0.03	0.03	0.03	0.06
δ	HE	0.8	0.5	0.5	0.5	0.5	0.8
	DE	0.88	0.46	0.42	0.43	0.46	0.88
$\alpha_{a}$	HE	0.7	0.6	0.5	0.5	0.6	0.7
	DE	0.75	0.60	0.55	0.55	0.60	0.75
$\beta_{a}$	HE	0.8	0.9	0.9	0.9	0.9	0.8
	DE	0.79	0.94	0.90	0.90	0.93	0.79

### A.3. Optimal Tuning of V, F and CR

In this Section we compare the performance of DE in terms of fitness, with different sizes of population *V*, and different values of the parameters *CR*, *F*. More specifically, the values considered are: V∈{10,20,30}, CR∈{0.2,0.4,0.6,0.8}, and F∈{0.4,0.8,1.6,2}. As a performance indicator, the average fitness of the population after 20 generations has been considered. Table A2 shows the indicator for *V* = 30 and for different values of *CR* and *F.* It is apparent that the values of *CR* and *F* do not affect significantly the performance. Table A3 shows the average number of generations to achieve 0.5 as average fitness of the population, over 5 trials, for different combinations of *CR* and *F*, and for the population size *V* = 30. According to the table, the values (*CR*, *F*) = (0.4, 0.8) cause faster convergence times of the tuning. This result is in line with other researches in the literature [42]. Finally, Figure A1 shows the average fitness of the population against the generation, for different values of *V*. It is apparent that higher values of *V*, up to 30 members, cause a significant reduction of the fitness. This result is in line with those presented in the literature. Thus, after these experiments the setting *V* = 30, *CR* = 0.4, and *F* = 0.8 has been considered.

**Table A2:** The average fitness of the population, over 5 trials, for *V* = 30, and for different combinations of *CR* and *F*.

*V* = 30		*CR*			
		0.2	0.4	0.6	0.8
*F*	0.4	0.545	0.478	0.494	0.551
	0.8	0.483	0.473	0.482	0.473
	1.2	0.487	0.475	0.474	0.476
	1.6	0.500	0.475	0.475	0.481
	2.0	0.492	0.483	0.475	0.487

**Table A3:** The average number of generations to converge to fitness 0.5, over 5 trials, for *V* = 30, and for different combinations of *CR* and *F*.

*V* = 30		*CR*			
		0.2	0.4	0.6	0.8
*F*	0.4	20.0	17.4	17.0	20.0
	0.8	18.8	16.8	17.4	18.0
	1.2	19.2	17.2	17.8	17.6
	1.6	19.0	18.0	17.6	17.6
	2.0	18.6	18.4	18.4	18.6
